# A potential immunotherapy target for breast cancer: parenchymal and immune-stromal expression of the NLRP3 inflammasome pathway

**DOI:** 10.1186/s12885-023-11609-4

**Published:** 2023-11-29

**Authors:** Qian-mei Zhu, Hui-xian Li, Pei-qing Ma, Lin-xin Wu, Tai-hang Wang, Wen-bin Li, Lin Zhang, Xue Yang, Xiangyi Kong, Yu-lin Sun, Tao Yan

**Affiliations:** 1 Department of Anesthesiology, Peking Union Medical College Hospital, Chinese Academy of Medical Sciences and Peking Union Medical College, Beijing, 100730 China; 2 Department of Anesthesiology, National Cancer Center/National Clinical Research Center for Cancer/Cancer Hospital, Chinese Academy of Medical Sciences and Peking Union Medical College, Beijing, 100021 China; 3 Department of Pathology, National Cancer Center/National Clinical Research Center for Cancer/Cancer Hospital, Chinese Academy of Medical Sciences and Peking Union Medical College, Beijing, 100021 China; 4 Suzhou Industrial Park Monash Research Institute of Science and Technology, Suzhou, China; 5 The School of Public Health and Preventive Medicine, Monash University, Victoria, Australia; 6 Department of Breast Surgical Oncology, National Cancer Center/National Clinical Research Center for Cancer/Cancer Hospital, Chinese Academy of Medical Sciences and Peking Union Medical College, Beijing, 100021 China; 7 State Key Laboratory of Molecular Oncology, National Cancer Center/ National Clinical Research Center for Cancer/Cancer Hospital, Chinese Academy of Medical Sciences & Peking Union Medical College, Beijing, 100021 China

**Keywords:** NLRP3 inflammasome, Innate immune system, Caspase-1, ASC, Breast cancer, Prognosis

## Abstract

**Background:**

The NOD-, LRR- and pyrin domain‑containing 3 (NLRP3) inflammasome is a critical component of the innate immune system. It has been known to play an important role in the carcinogenesis and prognosis of breast cancer patients. While the clinical evidence of the relationship between NLRP3 inflammasome activation and long-term survival is still limited, the possible roles of parenchymal or immune-stromal cells of breast cancer tissues in contributing to such carcinogenesis and progression still need to be clarified. This study is an analysis of patients receiving breast cancer surgery in a previous clinical trial.

**Methods:**

Immunohistochemistry (IHC) was used to detect the expression levels of NLRP3 inflammasome pathway-related proteins, including NLRP3, caspase-1, apoptosis-associated speck-like protein (ASC), IL-1β, and IL-18, in parenchymal and immune-stromal cells of breast cancer tissues compared to those of adjacent normal tissues, respectively. The relationship between NLRP3 inflammasome expression and clinicopathological characteristics, as well as 5-year survivals were analyzed using the Chi-square test, Kaplan–Meier survival curves, and Cox regression analysis.

**Results:**

In the parenchymal cells, ASC and IL-18 protein levels were significantly up-regulated in breast cancer tissues compared with adjacent normal tissues (*P*<0.05). In the immune-stromal cells, all the five NLRP3 inflammasome pathway-related proteins were significantly elevated in breast cancer tissues compared with adjacent normal tissues (*P* < 0.05). Carcinoma cell embolus was found to significantly correlate with high NLRP3 expression in parenchymal cells of the tumor (*x*^2^=4.592, *P*=0.032), while the expression of caspase-1 was negatively correlated with tumor progression. Histological grades were found to have a positive correlation with IL-18 expression in immune-stromal cells of the tumor (*x*^2^=14.808, *P*=0.001). Kaplan–Meier survival analysis revealed that high IL-18 expression in the immune-stromal cells and the positive carcinoma cell embolus were both associated with poor survival (*P* < 0.05). The multivariable Cox proportional hazards regression model implied that the high IL-18 expression and positive carcinoma cell embolus were both independent risk factors for unfavorable prognosis.

**Conclusions:**

The activation of NLRP3 inflammasome pathways in immune-stromal and tumor parenchymal cells in the innate immune system was not isotropic and the main functions are somewhat different in breast cancer patients. Caspase-1 in parenchymal cells of the tumor was negatively correlated with tumor progression, and upregulation of IL-18 in immune-stromal cells of breast cancer tissues is a promising prognostic biomarker and a potential immunotherapy target.

**Trial registration:**

This clinical trial has been registered at the Chictr.org.cn registry system on 21/08/2018 (ChiCTR1800017910)

**Supplementary Information:**

The online version contains supplementary material available at 10.1186/s12885-023-11609-4.

## Introduction

Breast cancer is one of the most common malignant tumors among women and carries different incidence and mortality rates among all ages [1]. Although the overall survival of breast cancer patients has improved due to early detection and treatment optimization, we still need to explore the mechanism of carcinogenesis to treat breast cancer more accurately. It has been known that tumorigenic immune reaction and inflammation contribute to the initiation and progression of breast cancer [2–4].

Inflammasomes are protein signaling complexes of immune-stromal cells and tumor cells that are in response to damage- and pathogen-associated molecular patterns (DAMPs and PAMPs) and trigger the release of inflammatory cytokines such as interleukin-1β (IL-1β) to participate in immune defense [5]. The NOD-, LRR- and pyrin domain‑containing 3 (NLRP3) inflammasome in the innate immune system, as the most concerned one of inflammasomes, consists of a NLRP3, an apoptosis-associated speck-like protein (ASC), and a pro-caspase-1 [6, 7]. The activated caspase-1 cleaves pro-IL-1β and pro-IL-18, and then the produced IL-1β and IL-18 are finally released to the outside of the cell membrane [8]. NLRP3 inflammasome pathway is closely associated with the occurrence and development of various kinds of cancers [9] and is also considered an important target for overcoming cancer [8]. When referring to breast cancer, basic studies have made some important hints indicating the NLRP3 inflammasome pathway as a possible therapeutic target for the prevention and treatment of breast cancer [10–13]. Very recently, a study began to evaluate the NLRP3 expression in breast cancer patients and found that higher expression of NLRP3 may predict a poor survival [14]. IL-1β was also reported to significantly influence the overall survival and distant metastasis of breast cancer [15, 16]. But such clinical studies are still very limited.

The initial published reports investigated the relationship between anesthetic/surgery techniques and pre-metastatic niche, as well as prognosis in the patients undergoing breast cancer surgery [17, 18]. In the current study, we convert to evaluate the differential expression and clinicopathological features of NLRP3 inflammasome pathway-related proteins, including NLRP3, caspase-1, ASC, IL-1β, and IL-18, in the tumor parenchymal and immune-stromal cells of breast cancer. And then the correlations between the levels of these proteins and the long-term survival of these breast cancer patients were also investigated.

## Methods

### Study design

This study is an analysis of patients receiving breast cancer surgery in a previous clinical trial. The primary trial endpoint has been published previously [17, 18]. This trial was a single-center, parallel-group, 1:1 randomized trial investigating the effect of anesthetic/surgery methods on the serum concentrations of Myeloid-derived suppressor cells(MDSCs), VEGF-C, TGF-β, as well as prognosis in the patients undergoing breast cancer surgery. Simple randomization was used to generate the random allocation sequence: participants were randomly allocated to treatment groups with equal probability using a random number generator. This prospective clinical trial was registered at the Chictr.org.cn registry system on 21/08/2018 (ChiCTR1800017910). Ethic approval was obtained from the Ethic Committee of Cancer Hospital (approval number: NCC2013YZ-06). The protocol was performed at Cancer Hospital, Chinese Academy of Medical Sciences and Peking Union Medical College from January 2016 to August 2016. The follow-ups were completed in July 2021.

### Patients

Eighty adult female patients aged 24 to 69 years, ASA physical status classified I to III, undergoing surgery for breast cancer were enrolled in the study. Surgery types included the mastectomy and breast-conserving surgery. General anesthesia included total intravenous anesthesia with propofol and inhalation anesthesia with sevoflurane. Other inclusion and exclusion criteria were detailed in the previous study [17]. All patients were informed of the relevant risks and signed informed consent.

### Immunohistochemistry (IHC) and IHC Scoring

Tissue sections (4 mm thick) were cut from formalin-fixed, paraffin-embedded blocks containing tumors and adjacent normal breast tissues for detecting NLRP3 inflammasome. The paraffin sections were put into xylene I, xylene II, and xylene III for 15 min respectively, then put into anhydrous ethanol I, anhydrous ethanol II, 85% alcohol, and 75% alcohol for 5 min respectively, and finally washed with distilled water. After antigen repair and blocking endogenous peroxidase, the tissue was covered with 3% BSA and sealed at room temperature for 30 min. The first antibody prepared by PBS in a certain proportion was dripped on the slices, and the slices were incubated overnight at 4 ° C in a wet box. The primary antibodies included anti-NLRP3 antibody (Abcam, ab214185), anti-IL-18 antibody (Abcam, ab243091), and anti-IL-1β antibody (Abcam, ab2105), anti-TMS1/ASC antibody (Abcam, ab180799), and anti-caspase-1 antibody (Abcam, ab62698). Then sections were rinsed three times using PBS. Tissues were incubated with goat anti-rabbit IgG H&L (Alexa Fluor® 488, Abcam, ab150077), the secondary antibody, at room temperature for 50 min. After adding the DAB solution, the color developing time was controlled under the microscope (Eclipse 80i, Nikon, Japan).

According to the staining intensity (IS), the score was divided into 4 grades: 0 (negative), 1 (light yellow), 2 (brownish yellow), and 3 (brownish brown). The proportion of positive cells was divided into 4 grades: 1 (≤ 25%), 2 (26%-50%), 3 (51%-75%) and 4 (> 75%). Multiply the two scores to obtain the final score result. The data obtained by multiplying the two scores represented the expression level, and 0-6 was identified as a low level and 7-12 was identified as a high level. NanoZoomer S210 (Hamamatsu, Japan) was used to scan the sections.

### Follow-up

Regular telephone follow-up was conducted every three months until 5 years after the operation. Survival time was calculated from the date of surgery to that of the last follow-up or death. Data regarding patient recurrence or death were got from inpatient and outpatient records, patients’ families, as well as local Public Security Census Register Office.

### Outcomes

The outcomes of this study included the expression levels of the NLRP3 inflammasome-related proteins in breast cancer tissues, the relationships between long-term survivals and NLRP3 inflammasome pathways expression in the breast cancer tissues, and the independent risk factors for patients postoperative survivals.

### Statistical analysis

The data in this study were analyzed using SPSS 23 software (SPSS Inc., New York, NY, USA). The chi-square test and Fisher exact test were applied for comparing between two groups. Kaplan–Meier analysis and log-rank test were used to analyze the relationship between the NLRP3 inflammasome expression levels in cancer tissues and the 5-year recurrence-free survival (RFS) and 5-year overall survival (OS) of breast cancer patients. Cox regression analysis was applied to determine risk factors for survival. A *P*-value of *<*0.05 was considered statistically significant.

## Results

### Clinicopathological characteristics

The clinicopathological characteristics of 80 breast cancer patients are summarized in Table 1, which were also enrolled in our previous study [17]. Among 80 patients, half were younger than 50 years and a half were older than 50 years. The tumor sizes of 37 patients were less than 2 cm and of 43 patients were larger than 2 cm. Seventy-four patients belonged to ASA I-II and 6 patients belonged to ASA III. The number of patients identified as TNM stage Tis, I, II, III were 3, 26, 33, and 18, respectively. While, the number of patients identified as histological grades I, II, III were 9, 45, and 26, respectively. Carcinoma cell embolus happened in 24 patients and nerve invasion was found in 70 patients. With respect to positive receptors, 59 patients were estrogen receptor positive (ER+), 57 patients were progesterone receptor positive (PR+) and 17 patients were human epidermal growth factor receptor 2 positive (HER-2+). Of the 80 patients enrolled, 10 patients belonged to triple-negative breast cancer (TNBC). Regarding tumor types, 3 were carcinoma in situ and 77 were invasive. Surgery types included mastectomy (49 patients) and breast-conserving surgery (31 patients). Anesthesia types included sevoflurane-based anesthesia (40 patients) and total intravenous anesthesia (TIVA) with propofol (40 patients).

**Table 1:** Clinicopathological characteristics of 80 breast cancer patients

**Characteristics**	**Number of patients**	**Percentage (%)**
**Total**	80	100
**Age**		
< 50 years	40	50.0
≥ 50 years	40	50.0
**ASA classification**		
I	48	60.0
II	26	32.5
III	6	7.5
**Tumor size (cm)**		
< 2	37	46.3
≥ 2	43	53.7
**TNM stage**		
Tis	3	3.7
I	26	32.5
II	33	41.3
III	18	22.5
**Histological grade**		
I	9	11.2
II	45	56.3
III	26	32.5
**Carcinoma cell embolus**		
Yes	24	30.0
No	56	70.0
**Nerve invasion**		
Yes	70	87.5
No	10	12.5
**Positive receptors**		
Estrogen	59	73.8
Progesterone	57	71.3
HER2	17	21.2
**TNBC**		
Yes	10	12.5
No	70	87.5
**Tumor type**		
Carcinoma in situ	3	3.7
Invasive carcinoma	77	96.3
**Surgery**		
Mastectomy	49	61.2
Breast conserving surgery	31	38.8
**Anesthesia**		
SEV	40	47.5
TIVA	40	52.5

*ASA* American Society of anesthesiologists, *TNM* Tumor node metastasis, *HER2* Human epidermal growth factor receptor 2, *TNBC* Triple-negative breast cancer, *SEV* sevoflurane-based anesthesia, *TIVA* Total intravenous anesthesia

### The expression levels of the NLRP3 inflammasome-related proteins in breast cancer tissues

**Fig. 1:**
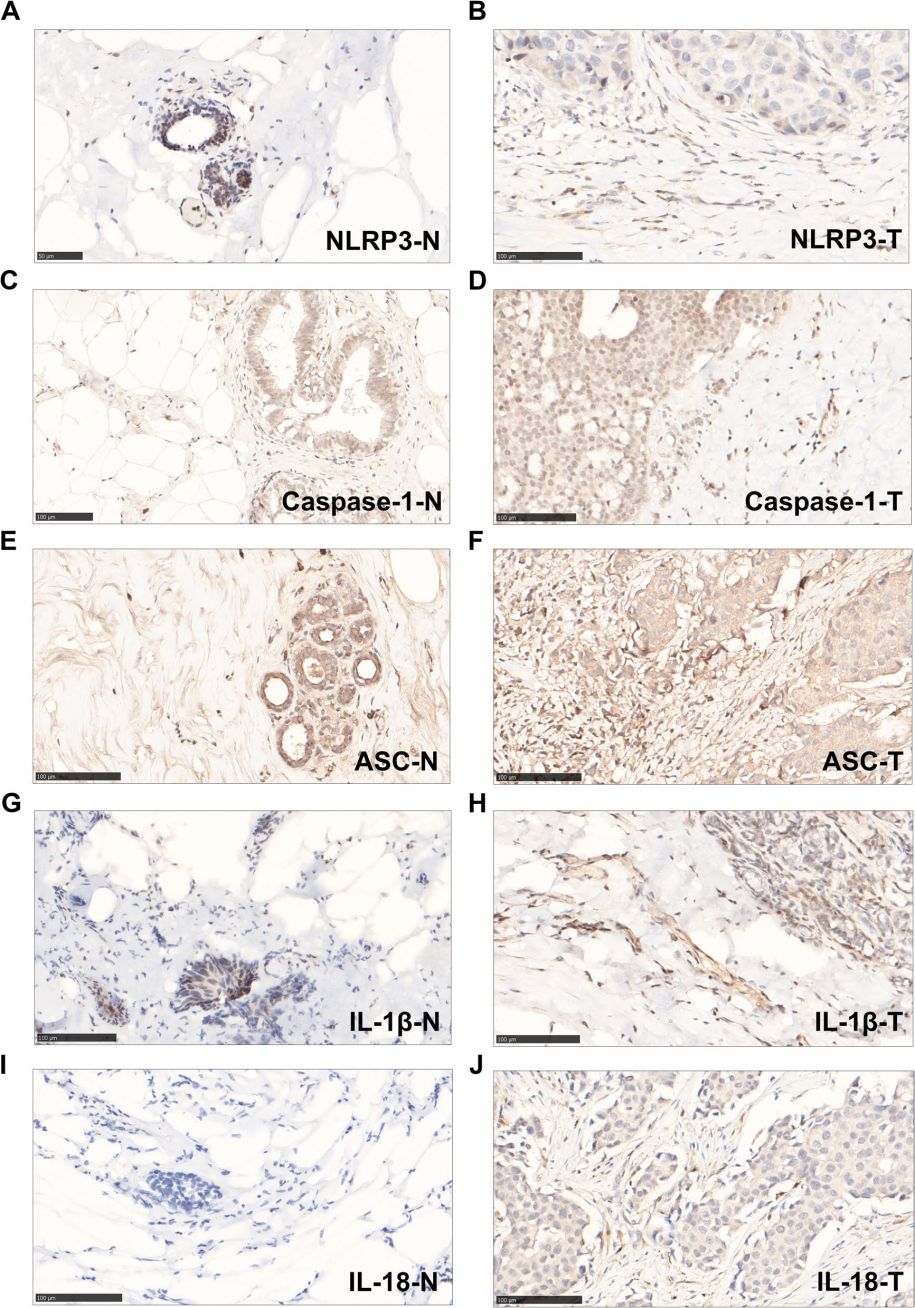
The immunohistochemical staining of NLRP3 inflammasome pathway related proteins in adjacent normal and breast cancer tissues of breast cancer patients. **A** NLRP3 expression in adjacent normal area (NLRP3-N); **B** NLRP3 expression in breast cancer area (NLRP3-T); **C** Caspase-1 expression in adjacent normal area (Caspase-1-N); **D** Caspase-1 expression in breast cancer area (Caspase-1-T); **E** ASC expression in adjacent normal area (ASC-N); **F** ASC expression in breast cancer area (ASC-T); **G** IL-1β expression in adjacent normal area (IL-1β-N); **H** IL-1β expression in breast cancer area (IL-1β-T); (**I**) IL-18 expression in adjacent normal area (IL-18-N); **J** IL-18 expression in breast cancer area (IL-18-T). NLRP3: NOD-, LRR- and pyrin domain‑containing 3, ASC: apoptosis-associated speck-like protein. Scale bar=100um

To further investigate the expression characteristics of NLRP3, caspase-1, ASC, IL-1β, and IL-18 proteins, immunohistochemistry assays were performed. We observed that these proteins were expressed in both the malignant cells and tumor stroma. NLRP3 (Fig. 1A-B), caspase-1 (Fig. 1C-D) and ASC (Fig. 1E-F) are mainly expressed in the cytoplasm; IL-1β (Fig. 1G-H) can be seen in the cytoplasm and extracellular matrix; IL-18 (Fig. 1I-J) can be seen in both cytoplasm and nucleus. Due to the absence of some specimens or the influence of staining technology, the number of staining of parenchymal cells and immune-stromal cells all decreased by 15 to 18. In the adjacent noncancerous tissues, the positive rates of NLRP3, caspase-1, ASC, IL-1β and IL-18 proteins in parenchyma were 66.7% (42/63), 73.0% (46/63), 73.0% (46/63), 68.3% (43/63) and 1.6% (1/64), respectively, whereas those in the stroma were 100.0% (62/62), 100.0% (64/64), 98.5% (64/65), 98.4% (62/63) and 93.7% (59/63), respectively. In comparison, in the tumor tissues, 84.4% (54/64), 74.6% (47/63), 93.7% (59/63), 73.0% (46/63) and 66.1% (41/62) of cases showed tumor cell-specific staining of NLRP3, caspase-1, ASC, IL-1β and IL-18 protein, respectively, whereas 100.0% (64/64), 100.0% (64/64), 100.0% (62/62), 100.0% (64/64) and 75.4% (49/65) of cases had positive immune-stromal staining.

**Fig. 2:**
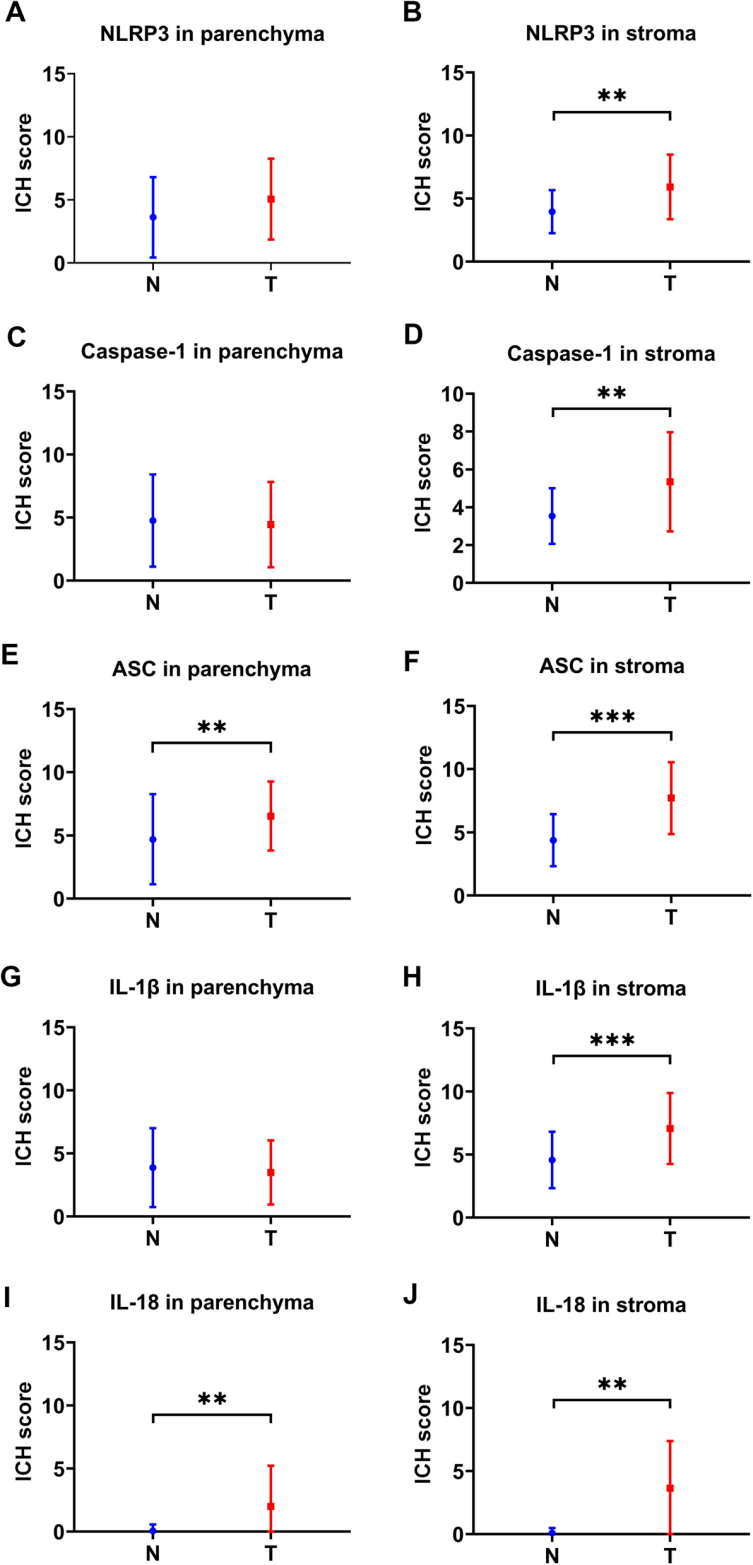
The immunohistochemical staining scores of NLRP3 inflammasome pathway proteins in parenchyma and stroma. **A** NLRP3 expression in parenchyma, between adjacent normal (N) area and breast cancer (T) area; **B** NLRP3 expression in stroma, between N and T area; **C** Caspase-1 expression in parenchyma, between adjacent normal (N) area and breast cancer (T) area; **D** Caspase-1 expression in stroma, between N and T area; breast cancer area; **E** ASC expression in parenchyma, between N and T area; **F** ASC expression in stroma, between N and T area; **G** IL-1β expression in parenchyma, between N and T area; **H** IL-1β expression in stroma, between N and T area; **I** IL-18 expression in parenchyma, between N and T area; **J** IL-18 expression in stroma, between N and T area; breast cancer area. NLRP3: NOD-, LRR- and pyrin domain‑containing 3, ASC: apoptosis-associated speck-like protein, IHC: immunohistochemistry. *: *P*<0.05, **: *P*<0.01, ***: *P*<0.001

As shown in Fig. 1A-B, there was no significant difference in the expression of NLRP3 between breast cancer tissue and adjacent normal tissue parenchyma cells (Fig. 2A); While, dramatically tumor immune-stromal overexpression of NLRP3 (*x*^2^=11.130, *P*=0.001; Fig. 2B) was found in breast cancer tissues. In Fig. 1C-D, no significant difference was seen in the expression of caspsae-1 between breast cancer tissue and adjacent normal tissue parenchyma cells (Fig. 2C); the immune-stromal overexpression of caspsae-1 (*x*^2^=11.549, *P*=0.001; Fig. 2D) was found in breast cancer tissues. In Fig. 1E-F, the expression levels of parenchymal ASC (*x*^2^=8.145, *P*=0.004; Fig. 2E) and stromal ASC (*x*^2^=24.303, *P*=0.000; Fig. 2F) were both significantly elevated in the tumor tissues compared with adjacent normal tissues. In Fig. 1G-H, no significant difference was seen in the expression of IL-1β between breast cancer tissue and adjacent normal tissue parenchyma cells (Fig. 2G); while the immune-stromal overexpression of IL-1β (*x*^2^=25.640, *P*=0.000; Fig. 2H) was found in breast cancer tissues. In Fig. 1I-J, the expression levels of parenchymal IL-18 (*x*^2^=8.818, *P*=0.003; Fig. 2I) and stromal IL-18 (*x*^2^=10.514, *P*=0.001; Fig. 2J) were both significantly elevated in the tumor tissues compared with adjacent normal tissues.

### Relationships between clinicopathological characteristics of the patients and the NLRP3 inflammasome pathways expression in the breast cancer tissues

To find the possible correlation between the expression of these five NLRP3 inflammasome pathway-related proteins in tumor tissues and patients’ clinicopathological characteristics, we separated all 5 molecular into low and high expression groups according to the IHC score. The chi-square test analysis revealed that in parenchymal cells of tumor (Table 2), carcinoma cell embolus (*x*^2^=4.592, *P*=0.032) was significantly correlated with high NLRP3 expression, and the expression level of caspase-1 was negatively correlated with tumor progression: small tumor size group (<2cm) showed higher caspase-1 expression and large tumor size group (≥2cm) showed lower caspase-1 expression (*x*^2^=9.979, *P*=0.002); both TNM grade III (*x*^2^=16.981, *P*=0.000) and histological grade III (*x*^2^=7.426, *P*=0.024) only accounted for 15% in the high caspase-1 group; patients with mastectomy, but not breast-conserving surgery, showed less caspase-1 expression in parenchymal cells (*x*^2^=5.962, *P*=0.015).

**Table 2:** Relationships between clinicopathological characteristics and the NLRP3 inflammasome pathways expression in the parenchyma of breast cancer tissues

**Characteristic**	**NLRP3**			**Caspase-1**			**ASC**			**IL-1β**			**IL-18**		
	**Low** (%)	**High** (%)	***P***	**Low** (%)	**High** (%)	***P***	**Low** (%)	**High** (%)	***P***	**Low** (%)	**High** (%)	***P***	**Low** (%)	**High** (%)	***P***
**Total**	41	23		43	20		22	41		54	9		54	8	
**Age**			0.102			0.409			0.420			0.198			0.182
< 50 years	18(43.9)	15(65.2)		21(48.8)	12(60.0)		10(45.5)	23(56.1)		26(48.1)	7(77.8)		31(57.4)	6(75.0)	
≥ 50 years	23(56.1)	8(34.8)		22(51.2)	8(40.0)		12(54.5)	18(43.9)		28(51.9)	2(22.2)		23(42.6)	2(25.0)	
**ASA classification**			0.597			0.050			0.538			0.451			0.098
I	24(58.5)	16(69.6)		25(58.1)	15(75.0)		12(54.5)	28(68.3)		33(61.1)	7(77.8)		37(68.5)	3(37.5)	
II	14(34.2)	5(21.7)		16(37.2)	2(10.0)		8(36.4)	10(24.4)		17(31.5)	1(11.1)		14(25.9)	3(37.5)	
III	3(7.3)	2(8.7)		2(4.7)	3(15.0)		2(9.1)	3(7.3)		4(7.4)	1(11.1)		3(5.5)	2(25.0)	
**Tumor size (cm)**			0.476			0.002			0.621			0.566			0.183
< 2	18(43.9)	8(34.8)		12(27.9)	14(70.0)		10(45.5)	16(39.0)		21(38.9)	5(55.6)		24(44.4)	1(12.5)	
≥ 2	23(56.1)	15(65.2)		31(72.1)	6(30.0)		12(54.5)	25(61.0)		33(61.1)	4(44.4)		30(55.6)	7(87.5)	
**TNM stage**			0.693			0.000			0.733			0.096			0.951
Tis	0	0		0	0		0	0		0	0		0	0	
I	12(29.3)	7(30.4)		6(14.0)	13(65.0)		8(36.4)	11(26.8)		14(25.9)	5(55.6)		16(29.6)	2(25.0)	
II	20(48.8)	9(39.2)		24(55.8)	4(20.0)		9(40.9)	19(46.4)		24(44.5)	4(44.4)		24(44.5)	4(50.0)	
III	9(21.9)	7(30.4)		13(30.2)	3(15.0)		5(22.7)	11(26.8)		16(29.6)	0		14(25.9)	2(25.0)	
**Histological grade**			1.000			0.024			0.668			0.088			0.325
I	2(4.9)	1(4.3)		1(2.8)	2(10.0)		0	3(7.3)		2(3.7)	2(22.2)		3(5.6)	0	
II	24(58.5)	13(56.5)		21(47.2)	15(75.0)		13(59.1)	23(56.1)		30(55.6)	5(55.6)		33(61.1)	3(37.5)	
III	15(36.6)	9(39.2)		21(50.0)	3(15.0)		9(40.9)	15(36.6)		22(40.7)	2(22.2)		18(33.3)	5(62.5)	
**Carcinoma cell embolus**			0.032			0.172			0.564			0.703			0.381
Yes	9(22.0)	11(47.8)		16(37.2)	4(20.0)		8(36.4)	12(29.3)		19(35.2)	2(22.2)		19(35.2)	1(12.5)	
No	32(78.0)	12(52.2)		27(62.8)	16(80.0)		14(63.6)	29(70.7)		35(64.8)	7(77.8)		35(64.8)	7(87.5)	
**Nerve invasion**			0.946			0.294			1.000			0.335			0.590
Yes	7(17.1)	3(13.0)		8(18.6)	1(5.0)		3(13.6)	6(14.6)		9(16.7)	0		9(16.7)	0	
No	34(82.9)	20(87.0)		35(81.4)	19(95.0)		19(86.4)	35(85.4)		45(83.3)	9(100.0)		45(83.3)	8(100.0)	
**Positive receptors**															
Estrogen	34	14		30	17		17	30		40	7		41	5	
Progesterone	32	14		29	16		15	30		38	7		40	5	
HER2	8	5		10	3		3	10		11	2		11	1	
**TNBC**			1.000			0.149			0.729			1.000			1.000
Yes	6(14.6)	4(17.4)		9(20.9)	1(5.0)		4(18.2)	6(14.6)		9(16.7)	1(11.1)		9(16.7)	1(12.5)	
No	35(85.4)	19(82.6)		34(79.1)	19(95.0)		18(81.8)	35(85.4)		45(83.3)	8(88.9)		45(83.3)	7(87.5)	
**Tumor type**			1.000			0.317			0.349			NA			1.000
Carcinoma in situ	1(2.4)	0		0	1(5.0)		1(4.5)	0		0	0		1(1.9)	0	
Invasive carcinoma	40(97.6)	23(100.0)		43(100.0)	19(95.0)		21(95.5)	41(100.0)		54(100.0)	9(100.0)		53(98.1)	8(100.0)	
**Surgery**			0.459			0.015			0.452			0.958			1.000
Mastectomy	27(65.9)	13(56.5)		31(72.1)	8(40.0)		15(68.2)	24(58.5)		34(63.0)	5(55.6)		34(63.0)	5(62.5)	
Breast conserving surgery	14(34.1)	10(43.5)		12(27.9)	12(60.0)		7(31.8)	17(41.5)		20(37.0)	4(44.4)		20(37.0)	3(37.5)	
**Anesthesia**			0.654			0.649			0.926			0.381			0.058
SEV	22(53.7)	11(47.8)		21(48.8)	11(55.0)		11(50.0)	21(51.2)		30(55.6)	3(33.3)		24(44.4)	7(87.5)	
TIVA	19(46.3)	12(52.2)		22(51.2)	9(45.0)		11(50.0)	20(48.8)		24(44.4)	6(66.7)		30(55.6)	1(12.5)	

Each molecular (NLRP3/ASC/IL-1β/IL-18) lacks 16 to 18 staining results for analysis. *NLRP3* NOD-, LRR- and pyrin domain‑containing 3, *ASC* Apoptosis-associated speck-like protein, *ASA* American Society of anesthesiologists, *TNM* Tumor node metastasis, *HER2* Human epidermal growth factor receptor 2, *TNBC* Triple-negative breast cancer, *SEV* Sevoflurane-based anesthesia, *TIVA* Total intravenous anesthesia, *NA* Not available

While in immune-stromal cells of the tumor (Table 3), ASA classification (*x*^2^=6.186, *P*=0.045) was found closely related to NLRP3 expression, and histological grades (*Fisher test, P*=0.001) were found closely related to IL-18 expression; and the proportion of high expression of ASC in the stroma of breast cancer in TNBC patients was significantly higher than that in non TNBC patients (*Fisher test, P*=0.015). That is ASA I group showed less NLRP3 expression, the histological grade III accounted for 90% in the high IL-18 group. The other features, such as age, nerve invasion, and anesthesia ways, did not present a significant correlation with NLRP3 inflammasome pathways expression (*P*>0.05).

**Table 3:** Relationships between clinicopathological characteristics and NLRP3 inflammasome pathways expression in stroma of breast cancer tissues

**Characteristic**	**NLRP3**			**Caspase-1**			**ASC**			**IL-1β**			**IL-18**		
	**Low** (%)	**High** (%)	***P***	**Low** (%)	**High** (%)	***P***	**Low** (%)	**High** (%)	***P***	**Low** (%)	**High** (%)	***P***	**Low** (%)	**High** (%)	***P***
**Total**	47	17		51	13		29	33		33	31		55	10	
**Age**			0.385			0.496			0.425			0.205			0.615
< 50 years	27(57.4)	7(41.2)		26(51.0)	8(61.5)		17(58.6)	16(48.5)		15(45.5)	19(61.3)		30(54.5)	4(40.0)	
≥ 50 years	20(42.6)	10(58.8)		25(49.0)	5(38.5)		12(41.4)	17(51.5)		18(54.5)	12(38.7)		25(45.5)	6(60.0)	
**ASA classification**			0.045			0.581			0.124			0.716			0.254
I	35(74.5)	7(41.2)		32(62.7)	10(76.9)		22(75.9)	19(57.5)		21(63.6)	21(67.7)		37(67.3)	5(50.0)	
II	10(21.3)	8(47.1)		15(29.4)	2(15.4)		4(13.8)	12(36.4)		10(30.3)	7(22.6)		15(27.3)	3(30.0)	
III	2(4.3)	2(11.7)		4(7.9)	1(7.7)		3(10.3)	2(6.1)		2(6.1)	3(9.7)		3(5.4)	2(20.0)	
**Tumor size (cm)**			0.635			0.114			0.874			0.762			0.249
< 2	19(40.4)	8(47.1)		19(37.3)	8(61.5)		12(41.4)	13(39.4)		14(42.4)	12(38.7)		25(45.5)	2(20.0)	
≥ 2	28(59.6)	9(52.9)		32(62.7)	5(38.5)		17(58.6)	20(60.6)		19(57.6)	19(61.3)		30(54.5)	8(80.0)	
**TNM stage**			0.178			0.087			0.497			0.767			0.265
Tis	0	0		0	0		0	0		0	0		0	0	
I	15(31.9)	5(29.4)		12(23.5)	7(53.8)		11(37.9)	8(24.2)		11(33.3)	9(29.0)		19(34.5)	1(10.0)	
II	17(36.2)	10(58.8)		25(49.0)	3(23.1)		11(37.9)	16(48.5)		13(39.4)	15(48.4)		23(41.8)	5(50.0)	
III	15(31.9)	2(11.8)		14(27.5)	3(23.1)		7(24.2)	9(27.3)		9(27.3)	7(22.6)		13(23.7)	4(40.0)	
**Histological grade**			0.348			0.759			0.558			0.544			0.001
I	4(8.5)	0		2(3.9)	0		2(6.9)	1(3.0)		3(9.1)	1(3.2)		3(5.5)	0	
II	28(59.6)	9(52.9)		29(56.9)	8(61.5)		18(62.1)	18(54.6)		19(57.6)	17(54.9)		37(67.2)	1(10.0)	
III	15(31.9)	8(47.1)		20(39.2)	5(38.5)		9(31.0)	14(42.4)		11(33.3)	13(41.9)		15(27.3)	9(90.0)	
**Carcinoma cell embolus**			0.341			0.860			0.847			0.730			0.591
Yes	17(36.2)	4(23.5)		17(33.3)	4(30.8)		9(31.0)	11(33.3)		12(36.4)	10(32.3)		19(34.5)	2(20.0)	
No	30(63.8)	13(76.5)		34(66.7)	9(69.2)		20(69.0)	22(66.7)		21(63.6)	21(67.7)		36(65.5)	8(80.0)	
**Nerve invasion**			1.000			0.102			0.351			0.536			1.000
Yes	7(14.9)	3(17.6)		9(17.6)	0		6(20.7)	3(9.1)		6(18.2)	3(9.7)		8(14.5)	1(10.0)	
No	40(85.1)	14(82.4)		42(82.4)	13(100.0)		23(79.3)	30(90.9)		27(81.8)	28(90.3)		47(85.5)	9(90.0)	
**Positive receptors**															
Estrogen	39	9		39	8		26	19		27	20		43	5	
Progesterone	38	8		30	7		25	19		26	20		41	5	
HER2	8	6		11	3		6	7		9	5		10	3	
**TNBC**			0.230			0.411			0.015			0.178			0.175
Yes	5(10.6)	4(23.5)		7(13.7)	3(23.1)		1(3.4)	9(27.3)		3(9.1)	7(22.6)		7(12.7)	3(30.0)	
No	42(89.4)	13(76.5)		44(86.3)	10(76.9)		28(96.6)	24(72.7)		30(90.9)	24(77.4)		48(87.3)	7(70.0)	
**Tumor type**			1.000			0.611			0.468			NA			1.000
Carcinoma in situ	1(2.1)	0		1(2.0)	0		1(3.4)	0		0	0		1(1.8)	0	
Invasive carcinoma	46(97.9)	17(100.0)		50(98.0)	13(100.0)		28(96.6)	33(100.0)		33(100.0)	31(100.0)		54(98.2)	10(100.0)	
**Surgery**			0.430			0.063			0.522			0.220			0.807
Mastectomy	30(63.8)	9(52.9)		34(66.7)	5(38.5)		19(65.5)	19(57.6)		23(69.7)	17(54.8)		33(60.0)	7(70.0)	
Breast-conserving surgery	17(36.2)	8(47.1)		17(33.3)	8(61.5)		10(34.5)	14(42.4)		10(30.3)	14(45.2)		22(40.0)	3(30.0)	
**Anesthesia**			0.317			0.756			0.799			0.313			0.328
SEV	21(44.7)	10(58.8)		26(51.0)	6(46.2)		15(51.7)	16(48.5)		15(45.5)	18(58.1)		26(47.3)	7(70.0)	
TIVA	26(55.3)	7(41.2)		25(49.0)	7(53.8)		14(48.3)	17(51.5)		18(54.5)	13(41.9)		29(52.7)	3(30.0)	

Each molecular (NLRP3/caspase-1/ASC/IL-1β/IL-18) lacks 15 to 18 staining results for analysis. *NLRP3* NOD-, LRR- and pyrin domain‑containing 3, *ASC* Apoptosis-associated speck-like protein, *ASA* American Society of anesthesiologists, *TNM* Tumor node metastasis, HER2: human epidermal growth factor receptor 2, *TNBC* triple-negative breast cancer, *SEV* Sevoflurane-based anesthesia, *TIVA* Total intravenous anesthesia, *NA* Not available

### Relationships between long-term survivals and NLRP3 inflammasome pathways expression in the breast cancer tissues

**Fig. 3:**
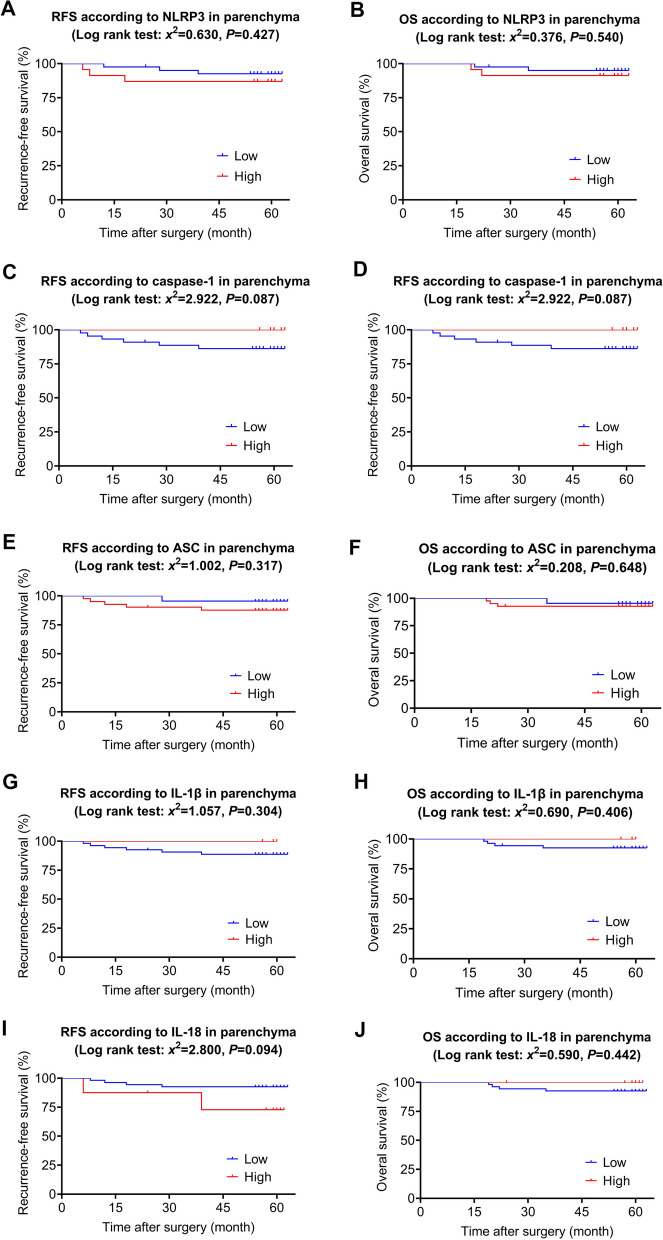
Kaplan–Maier curve analysis and log-rank test for the relationships between long-term survivals and NLRP3 inflammasome pathways expression in the parenchyma of breast cancer tissues. **A** RFS according to NLRP3 low versus high patients; **B** OS according to NLRP3 low versus high patients; **(** RFS according to caspase-1 low versus high patients; **D** OS according to caspase-1 low versus high patients; **E** RFS according to ASC low versus high patients; **F** OS according to ASC low versus high patients; **G** RFS according to IL-1β low versus high patients; **H** OS according to IL-1β low versus high patients; **I** RFS according to IL-18 low versus high patients; **J** OS according to IL-18 low versus high patients. According to the staining intensity (IS), the score 0-6 was identified as the low level and 7-12 was identified as the high level. NLRP3: NOD-, LRR- and pyrin domain‑containing 3, ASC: apoptosis-associated speck-like protein, RFS: recurrence-free survival, OS: overall survival

**Fig. 4:**
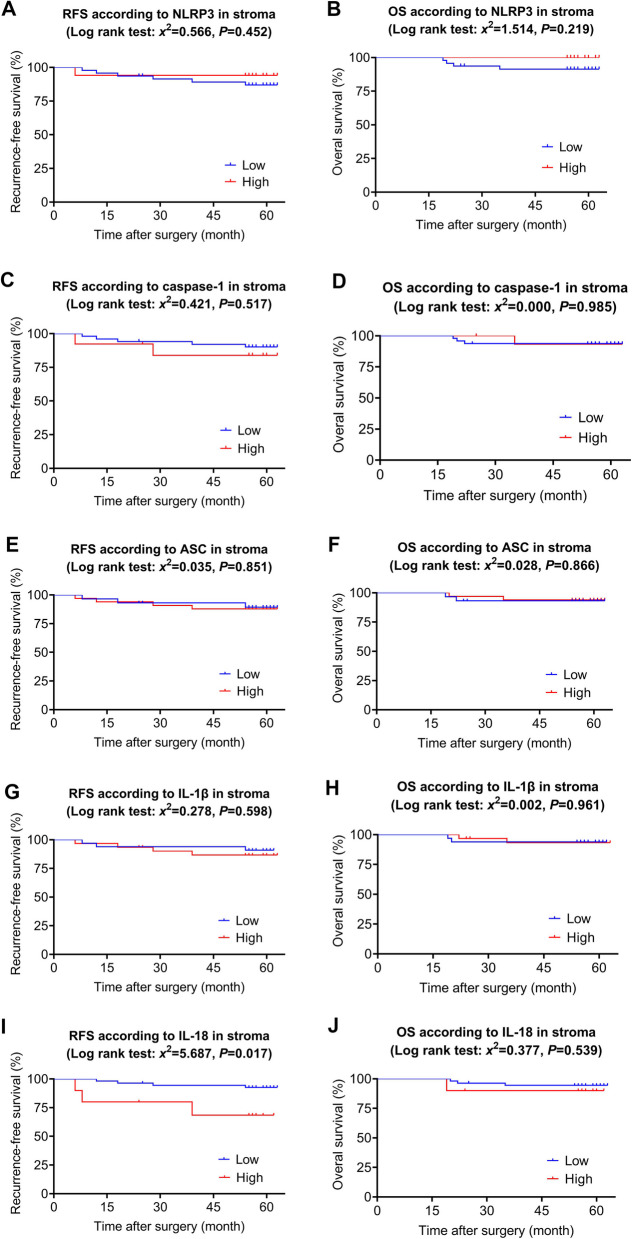
Kaplan–Maier curve analysis and log-rank test for the relationships between long-term survivals and NLRP3 inflammasome pathways expression in stroma of breast cancer tissues. **A** RFS according to NLRP3 low versus high patients; **B** OS according to NLRP3 low versus high patients; **C** RFS according to caspase-1 low versus high patients; **D** OS according to caspase-1 low versus high patients; **E** RFS according to ASC low versus high patients; **F** OS according to ASC low versus high patients; **G** RFS according to IL-1β low versus high patients; **H** OS according to IL-1β low versus high patients; **I** RFS according to IL-18 low versus high patients; **J** OS according to IL-18 low versus high patients. According to the staining intensity (IS), the score 0-6 was identified as the low level and 7-12 was identified as the high level. NLRP3: NOD-, LRR- and pyrin domain‑containing 3, ASC: apoptosis-associated speck-like protein, RFS: recurrence-free survival, OS: overall survival

The 80 patients were followed up for 5 years after surgery. The expression levels of NLRP3 inflammasome pathways were all divided into low and high groups. In the parenchymal cells, Kaplan–Meier survival analysis revealed that there were no significant differences in 5-year survival between the high expression group and low expression group of these NLRP3 inflammasome pathways (Fig. 3, *P*>0.05). In the immune-stromal cells, we found that the expression level of NLRP3 (Fig. 4A-B, *P*>0.05), caspase-1(Fig. 4C-D, *P*>0.05), ASC (Fig. 4E-F, *P*>0.05) and IL-1β (Fig. 4G-H, *P*>0.05) did not significantly affect the 5-year survival. While the high expression group of IL-18 indicated a poor 5-year RFS than that in the low expression group (Fig. 4I, *x*^*2*^=5.687, *P*=0.017), without affecting the 5-year OS (Fig. 4J, *P*>0.05). In summary, high expression of IL-18 in breast cancer stromal area is associated with poor survival in breast cancer patients after surgery.

### The analysis of independent risk factors for long-term survivals

Kaplan–Meier survival analysis also revealed that when referring to the clinicopathological characteristics, only positive carcinoma cell embolus was significantly associated with worse RFS (Supplemental Fig. 1F, *x*^*2*^=4.557, *P*=0.033) and worse OS (Supplemental Fig. 1F, *x*^*2*^=3.986, *P*=0.046). Tumor size tend to associate with worse OS, although it is not statistically significant (Supplemental Fig. 1C, *x*^*2*^=3.566, *p*=0.059). Other characteristics, including age, ASA classification, tumor size and tumor type, TNM stage, histological grade, nerve invasion, targeting receptors, anesthesia, and surgery types, present no significant correlations with prognosis.

For multivariable analysis, the distribution of IL-18 in the immune-stromal cells and carcinoma cell embolus was assessed by the Cox proportional-hazards model (Table 4). The results showed that IL-18 in the immune-stromal cells and carcinoma cell embolus were the independent risk factors influencing the 5-year RFS of breast cancer patients. Compared with the low expression group, patients in the high IL-18 expression group had lower 5-year RFS after surgery (*P*=0.004, HR=34.73, 95%CI: 3.05-395.73). Compared with the negative carcinoma cell embolus group, patients in the positive group had lower 5-year RFS after surgery (*P*=0.008, HR=29.81, 95%CI: 2.46-361.90). IL-18 and carcinoma cell embolus did not significantly influence the 5-year OS in this study.

**Table 4:** Multivariable Cox regression analysis of long-term survival

**Characteristics**	**RFS**			**OS**		
	**HR**	**95% CI**	***P***	**HR**	**95% CI**	***P***
**IL-18**	34.73	3.05-395.73	0.004	3.53	0.34-36.88	0.292
**Carcinoma cell embolus**	29.81	2.46-361.90	0.008	6.45	0.67-62.03	0.106

*RFS* Recurrence-free survival, *OS* Overall survival, *CI* Confidence interval, *HR* Hazard ratio

## Discussion

In tumor tissues, the tumor microenvironment (TME) composed of cancer cells and immune-stromal cells plays a major biological role and is closely related to tumor occurrence, growth, and metastasis [19]. Immune-stromal cells are considered to include fibroblasts, endothelium of blood vessels, and inflammatory and immune cells. In breast cancer tissues, cancer-associated fibroblasts (CAFs) and tumor-associated macrophages (TAMs) are both the prominent inflammatory stimulators in the stroma that contribute to the TME [7, 20, 21].

In the TME, the dysregulation of NLRP3 inflammasome activation is supposed to promote the development of all stages of tumorigenesis, although the inflammatory cells continuously change their phenotypic and functional characteristics throughout the whole process [19]. In breast cancer, the NLRP3 inflammasome pathway is closely related to tumor proliferation, angiogenesis, and invasiveness [22, 23]. NLRP3 inflammasome activation followed by the caspase-1-dependent release of pro-inflammatory cytokines IL-1β and IL-18 leads to the development of acute and chronic inflammation [7, 24]. Such persistent inflammatory response will induce the epithelial-to-mesenchymal transition, influence cellular plasticity, engender cancer stem cells, and interfere with immune cells entering the TME and playing immune function [7, 25, 26]. There is another inflammatory form of programmed gasdermin D-mediated cell death that closely associated with NLRP3 activation: pyroptosis, which is characterized by cellular swelling and rupture, lysis, nuclear condensation, as well as IL-1β and IL-18 leakage [7, 27]. Pyroptosis also helps to induce progressive NLRP3 inflammasome activation by releasing DAMPs [28–30], but the lytic and immunogenic nature of pyroptosis ensures to demising cancer cells and containing cancer progression by immune resistance effect [31]. The current evidence suggests that the activation of the NLRP3 inflammasome pathway may be pros and cons in the development of breast cancer.

Several previous studies had investigated the location and expression characteristics of NLRP3 inflammasome in breast cancer tissues. It has been broadly reported that the increased expression of NLRP3 inflammasome in breast CAFs and TAMs, the two major cells in the stroma of breast tumor, contribute to tumor progression and metastasis of breast cancer patients [12, 32, 33]. Through searching for the NCBI GEO dataset, the expression of *NLRP3*, *PYCARD*, *CASP1*, and *IL-1β* genes in the tumor-associated stroma of breast cancer patients were found to be increased with the pathological stage when compared with normal breast stroma [12]. A recent study just focused on NLRP3 inflammasome activation in the tumor parenchymal counterparts compared with non-cancerous counterparts [34]. They found that NLRP3 and ASC proteins were significantly activated in invasive ductal carcinoma cells and patients with higher NLRP3 expression acted out worse 5-year DFS. In addition, an increased level of NLRP3 has also been affirmed in several breast cancer cell lines in vitro and promotes tumor growth [13, 14, 35, 36]. Another work showed that NLRP3 inflammasome pathway-related genes were all aberrantly expressed in breast cancer tissues without distinguishing parenchymal or immune-stromal expression, and *NLRP3* showed a high frequency of copy number variation and higher expression [37]. After analyzed by univariate cox regression analysis, only *IL-18* was found a protective factor for better survival outcomes for breast cancer patients. However, there is so far no systematic comparative observation of the expression characteristics and prognostic significance of these proteins in both the parenchymal and immune-stromal parts of breast cancer patients.

In our study, we separately analyzed the expression levels of NLRP3 inflammasome pathway-related proteins in immune-stromal and tumor parenchymal cells. Without distinguishing between fibroblasts and macrophages, we found that NLRP3, caspase-1, ASC, IL-1β, and IL-18 were all elevated in the breast cancer immune-stromal cells. While in parenchymal cells, only ASC and IL-18 were significantly upregulated when compared with normal tissues. That is, there was no significant change in the protein level of NLRP3 derived from tumor parenchyma cells. But as previously reported, NLRP3 and ASC proteins were both significantly upregulated in invasive ductal carcinoma cells of breast cancer patients [34]. We may consider the following reasons for such differential results of NLRP3 expression: We used a scoring system to assess the visual intensity of IHC staining and the proportion of positive cells, which is a semi-quantitative analysis as opposed to using quantitative detection methods such as PCR or Western blot; we involved fewer samples than the previous study did; from the clinical characteristics, the different proportions of tumor grade and the positive receptor may be the reasons for the difference between the two studies; ethnic differences in Asia and Europe can also have an impact.

IL-18, as a proinflammatory cytokine widely produced by myeloid cells, epithelial cells and fibroblasts, plays both pro- and anti-tumorigenic roles, and it can be easily detected in solid tissues and peripheral blood [38–40]. As we have observed in the present study, IL-18 protein was expressed as low as 1.6% in adjacent normal parenchyma, whereas it was found in 93.7% of the stroma. Besides, the elevated protein level of IL-18 in the immune-stromal cells of cancer tissues, rather than tumor cells, was found closely associated with poor 5-year RFS, which indicates that IL-18 may be a key target for improving the long-term prognosis of breast cancer patients in the future. While one study recommended that high *IL-18* gene expression detected in breast cancer tissues was a protective factor for breast cancer prognosis [37]. But as far as we know, the higher serum IL‐18 level is associated with worse postoperative prognosis in patients with breast cancer [41, 42]. And breast cancer cell-derived IL-18 also predicts a bad prognosis in patients with TNBC by increasing the immunosuppressive CD56dimCD16dim/- NK cell fraction and inducing PD-1 expression on NK cells [43]. The previous studies have not addressed the cellular source of IL-18, and our elucidation of its stromal localization establishes a basis for future functional investigations. Even if the pro‐and anti‐tumorigenic mechanism of IL-18 coexist and is debated continuously, the tumor cell-derived and immune-stromal cell-derived IL-18 would predict a poor prognosis in breast cancer patients.

In addition, according to a previous study, the caspase-1 gene in breast cancer tissues was significantly decreased compared with the adjacent normal tissues [44]. Our study found that caspase-1 expressed in the parenchymal tumor cells was negatively correlated with tumor progression, for example, higher caspase-1 means smaller tumor size and lower invasive grade. While caspase-1 was significantly upregulated in immune-stromal cells of cancer tissues than that of normal tissues. It was known that after being activated by NLRP3, caspase-1 participates in initiating the cell death process by cleaving GSDMD, a member of gasdermins (GSDMs) in humans [7]. Thus, it can be inferred that the activation of NLRP3 and caspase-1 in tumor parenchymal cells are mainly involved in pyroptosis and inhibit tumor growth of breast cancer, while the activation in tumor immune-stromal cells mainly contributes to inflammatory reaction and promote tumor progression.

There are still many deficiencies in this study. For example, the number of involved patients is small, and some of the specimens had been missed, the conclusion may be identified on a more large scale in the future. Due to the small number of cases involved, there was no subgroup analysis of tumor pathological classification. At present, the TNBC is the most malignant one and researchers are more interested in it [45, 46]. In addition, tumor immune-stromal cells were not specifically classified in this study.

In conclusion, our study reveals that the activation of NLRP3 inflammasome pathways in immune-stromal and tumor parenchymal cells were not isotropic and the main functions are somewhat different in breast cancer patients. Caspase-1 in parenchymal cells of the tumor was negatively correlated with tumor progression, and upregulation of IL-18 in immune-stromal cells of breast cancer tissues is a potential immunotherapy target and a promising prognostic biomarker in this study. The innovative discovery of the study, which differs from previous research, is that elevated IL-18 expression in breast cancer stromal cells, rather than tumor cells, correlates with an unfavorable prognosis. Different cellular sources and distribution often correlate with different functions, indicating the possibility of a more complex role for IL-18 in the tumorigenesis and development of breast cancer. Further studies are still needed to explore the close relationship between NLRP3 inflammasome and the post-operative long-term prognosis of breast cancer patients.

### Supplementary Information

**Additional file 1: Supplementary Figure 1.** 

## Data Availability

The datasets supporting the conclusions of this article are available if contacting the corresponding author for a data request.
